# Isolated Medial Rectus Palsy in a Patient After Percutaneous Transluminal Coronary Angioplasty

**DOI:** 10.7759/cureus.29653

**Published:** 2022-09-27

**Authors:** Avi Sharma, Sachin Daigavane

**Affiliations:** 1 Department of Ophthalmology, Jawaharlal Nehru Medical College, Datta Meghe Institute of Medical Sciences, Wardha, IND

**Keywords:** neurophthalmology, ophthalmology, ptca, radiology, medial rectus palsy

## Abstract

Diplopia, a very common ophthalmic complaint, is a potential first sign of severe pathology. Here, we present a case of an atypical midbrain infarction targeting the lateral subnucleus of the oculomotor nuclear complex that manifested as diplopia with no additional symptoms of a stroke episode. Axial diffusion-weighted and coronal T2-weighted magnetic resonance imaging showed an infarct in the rostral midbrain affecting the subnucleus of the medial rectus located ventrally. Diffusion-weighted imaging was used to diagnose the medial rectus nucleus infarct.

## Introduction

Extraocular muscle palsy is a sign of an underlying neurological issue, which is linked to systemic ailments such as diabetes, hypertension, hypercholesterolemia, and heart conditions [1]. Oculomotor nerve palsy may be caused by vascular disorders of the midbrain and falls into two categories, namely, nuclear and infranuclear. Bilateral ptosis and contralateral elevation palsy are associated with the nuclear type [2-4]. Oculomotor nerve palsy resulting from infranuclear nerve damage varies in severity, for example, partial oculomotor nerve palsy can occur [5]. Usually, isolated unilateral extraocular palsies are associated with orbital lesions or muscular disorders.

Isolated medial rectus palsy is an extremely rare presentation in clinical practice, particularly when no other stroke manifestations are identified. Diplopia is a subjective complaint that requires thorough evaluation for management. Identifying the cause of diplopia can be challenging due to the large number of differential diagnoses that exist. In this case report, we present a case of midbrain infarction that presented as isolated medial rectus palsy with sudden onset of diplopia post-percutaneous transluminal coronary angioplasty (PTCA).

## Case presentation

Patient details

A 66-year-old woman reported with chief complaints of abrupt-onset diplopia that had been present for two days to the Department of Ophthalmology. It was not associated with drooping of eyelids. There was no previous history of painful ocular movements, colored halos, or visual field loss. No history of any other neurological symptoms such as numbness or tingling, one-sided weakness, or slurred speech was reported. She was a postoperative case of PTCA of the right coronary artery (RCA) done six days ago. She was a known case of hypertension on tablet atenolol 50 mg PO OD. There was no history of illicit drug abuse, smoking, or alcohol consumption.

Clinical examination

**Figure 1:**
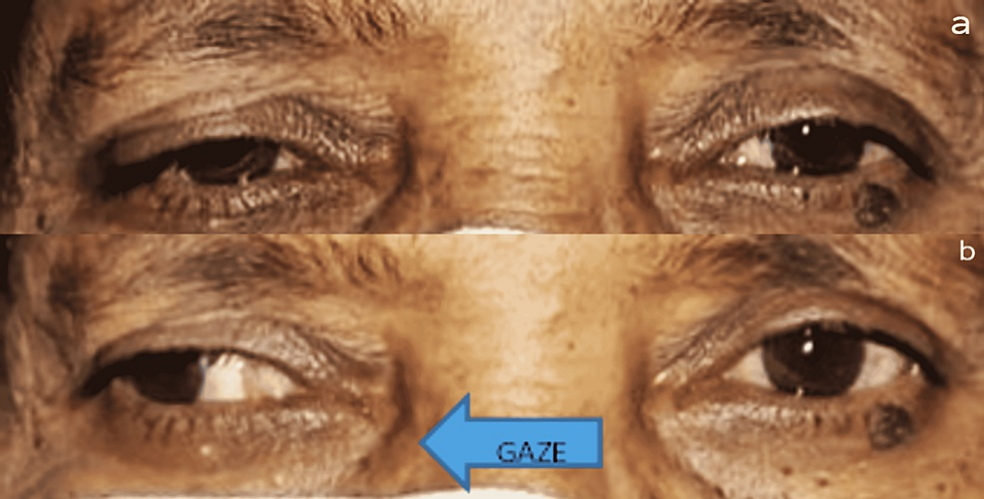
(a) Normal Hirshberg test. (b) Restricted adduction seen in the left eye when the patient is gazing toward the right.

On ophthalmological examination, the unaided visual acuity was 6/24P in both eyes with improvement to 6/18 with pinhole. With -1.50 DS refractive correction in the right eye and -1.25 DS refractive correction in the left eye, the best-corrected visual acuity (BCVA) was 6/9 in both eyes, with N6 near vision with a +3.00 DS addition. Hirschberg test was orthophoric (Figure 1a). Examination of the right eye did not reveal any abnormality. The slit lamp examination was within normal limits for the left eye. Pupils were equal in size, round in shape, reactive to light, and accommodated. Relative afferent pupillary defect (RAPD) was absent. Intraocular pressure was within normal limits. The anterior-segment evaluation was normal. Examination of other cranial nerves was within normal limits. Lens status was pseudophakia in both eyes. On examination of extraocular movements, complete restriction of movement was noted on adduction of the left eye (Figure 1b). Diplopia charting demonstrated uncrossed diplopia.

On posterior-segment examination, a clear media, the optic disc with all margins well defined, a cup-disc ratio of 0.3:1, no hyperemic disc with blood vessels, and normal macula with bright foveal reflex were seen in both eyes. On a systemic assessment, there were no further indicators of neurological deficiency. It was diagnosed that the left eye had medial rectus palsy (paralytic exotropia).

**Figure 2:**
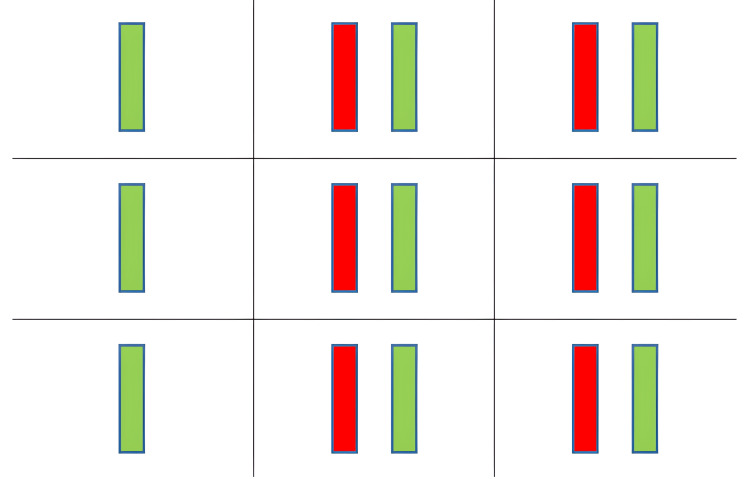
Diplopia charting showing uncrossed diplopia. Red: view from the right eye. Green: view from the left eye.

Imaging

**Figure 3:**
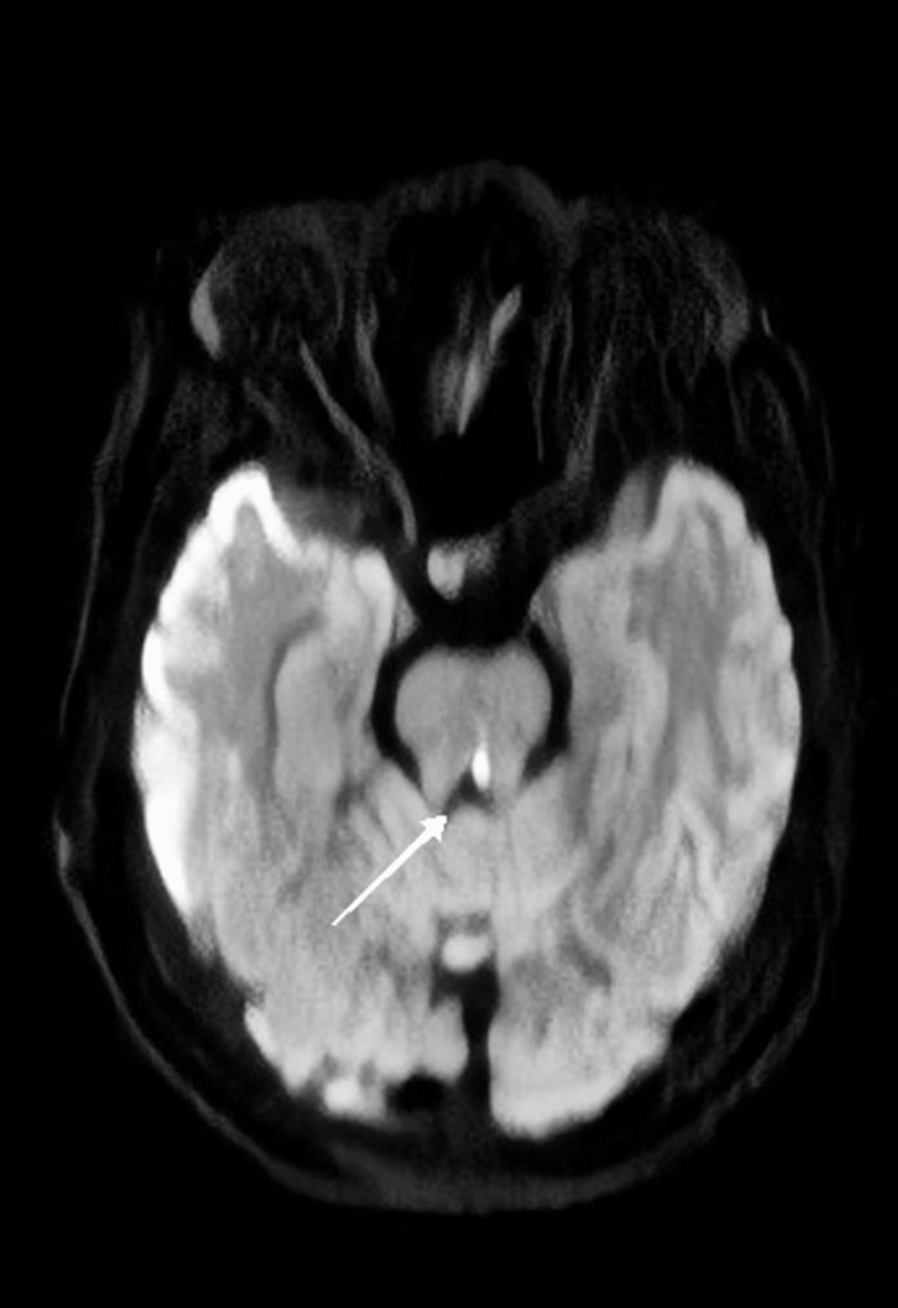
Magnetic resonance imaging of the brain reveals a focus of hyperintensity in the midbrain suggestive of acute infarction (white arrow).

Magnetic resonance imaging (MRI) of the brain showed a few, tiny ill-defined altered signal intensity areas in the right parietal region, lentiform nucleus, thalamus, and left cerebral peduncle appearing hyperintense on T2/fluid-attenuated inversion recovery, iso-to-hypointense on T1-weighted imaging (T1WI) (Figure 3). Restriction on diffusion-weighted imaging (DWI) and no blooming on gradient-recalled echo were suggestive of acute infarct in the above-mentioned areas. These were suggestive of multiple tiny acute infarcts (embolic) in the right parietal region, lentiform nucleus, thalamus, and left cerebral peduncle.

## Discussion

The cranial nerve III nuclei are located in the midbrain, in the dorsal region beneath the third and the fourth ventricles. The cranial nerve III nucleus is a complex of nuclei that includes four unpaired subnuclei for the four extraocular muscles (superior rectus, inferior rectus, medial rectus, and inferior oblique), a single subnucleus called the central caudal nucleus for the levator palpebrae superioris muscle along with two Edinger Westphal nuclei for the constrictor pupillae muscles [6].

Subnuclei for the ipsilateral inferior rectus are located ventrally to the superior rectus subnuclei, followed by intermediate subnuclei for the ipsilateral inferior oblique, and subnuclei for the ipsilateral medial rectus are located most ventrally. Innervating fibers exit ventrally ipsilateral to where they originate in the levator palpebrae superioris, medial rectus, inferior rectus, pupil sphincter, and ciliary body muscles. Conversely, fibers that exit the brainstem from the superior rectus subnucleus, located along the midline, cross before innervating the superior rectus muscle.

Although partial oculomotor nerve palsy was seen in our case, it was of the pupil-sparing type. The complete absence of ptosis and nystagmus helped us rule out other differentials of muscle palsy such as compressive lesions, posterior communicating artery aneurysms, or internuclear ophthalmoplegia. The age of the patient and her systemic illness made midbrain infarction causing isolated medial rectus palsy a more likely diagnosis. We confirmed the diagnosis on MRI.

In the medical literature, only 59 occurrences of isolated medial rectus palsy have been documented, with the majority of them being single case presentations. This indicates how uncommon isolated medial rectus palsy presentations are in clinical practice [7-17]. Unilateral ocular palsy is typically associated with orbital lesions or muscular illnesses, and seldom with a nuclear lesion of the III nerve, while inferior oblique muscle palsy due to intermediate subnuclei lesions is more prevalent [18]. The loss of adduction of the medial rectus of the left eye, supplied by the lateral subnucleus inside the oculomotor nerve nuclear complex, is evident in our case.

We report an atypical case of nuclear oculomotor nerve palsy with an isolated infarct involving the subnuclei of the medial rectus in the rostral midbrain, located ventrally. This was detected with DWI [1]. The patient was managed conservatively and started on antiplatelet therapy and monitored closely on follow-up.

## Conclusions

Oculomotor nerve palsy, whether isolated or predominant, is a rare complication of PTCA. Early neuroimaging is crucial for detecting an infarct lesion, and DWI aids in the detection of ischemic nuclear lesions. DWI has a higher sensitivity than traditional T1/T2 MRI as it might not be able to detect an infarct for up to six hours, but an enhanced DWI signal in ischemic brain tissue can be noticed minutes after artery occlusion. The incidence of detecting a midbrain infarct leading to isolated oculomotor palsies has increased with the use of DWI along with new multimodality, MRI. A unilateral medial rectus nuclear palsy is often the only manifestation of a midbrain infarction. A systemic clinical approach supplemented by appropriate history and examination is essential for elucidating the cause of diplopia and preventing missed serious causes such as cerebrovascular accidents There is a need to study the anatomical proclivity for the ventral part of III cranial nerve nucleus to have an infarct as there have been a few other cases similar to ours recorded.
